# Access to hepatitis C medicines

**DOI:** 10.2471/BLT.15.157784

**Published:** 2015-10-08

**Authors:** Danny J Edwards, Delphi GM Coppens, Tara L Prasad, Laurien A Rook, Jayasree K Iyer

**Affiliations:** a Access to Medicine Foundation, Scheepmakersdijk 5A, 2011 AS, Haarlem, Netherlands.

## Abstract

Hepatitis C is a global epidemic. Worldwide, 185 million people are estimated to be infected, most of whom live in low- and middle-income countries. Recent advances in the development of antiviral drugs have produced therapies that are more effective, safer and better tolerated than existing treatments for the disease. These therapies present an opportunity to curb the epidemic, provided that they are affordable, that generic production of these medicines is scaled up and that awareness and screening programmes are strengthened. Pharmaceutical companies have a central role to play. We examined the marketed products, pipelines and access to medicine strategies of 20 of the world’s largest pharmaceutical companies. Six of these companies are developing medicines for hepatitis C: AbbVie, Bristol-Myers Squibb, Gilead, Johnson & Johnson, Merck & Co. and Roche. These companies employ a range of approaches to supporting hepatitis C treatment, including pricing strategies, voluntary licensing, capacity building and drug donations. We give an overview of the engagement of these companies in addressing access to hepatitis C products. We suggest actions companies can take to play a greater role in curbing this epidemic: (i) prioritizing affordability assessments; (ii) developing access strategies early in the product lifecycle; and (iii) licensing to manufacturers of generic medicines.

## Résumé

L'hépatite C est une épidémie mondiale. On estime à 185 millions le nombre d'individus infectés par ce virus dans le monde, la plupart vivant dans des pays à revenu faible ou intermédiaire. Les récents progrès dans la mise au point de médicaments antiviraux ont conduit à des traitements plus efficaces, plus surs et mieux tolérés que les traitements existants pour soigner cette maladie. Ces traitements permettraient de freiner l'épidémie, à condition que leur coût soit abordable, que la production de médicaments génériques soit intensifiée et que les programmes de sensibilisation et de dépistage soient renforcés. Les sociétés pharmaceutiques ont, à cet égard, un rôle central à jouer. Nous avons examiné les produits commercialisés, les produits à l'étude et les stratégies d'accès aux médicaments de 20 des plus importantes sociétés pharmaceutiques mondiales. Six de ces sociétés développent des médicaments pour soigner l'hépatite C: AbbVie, Bristol-Myers Squibb, Gilead, Johnson & Johnson, Merck & Co. et Roche. Ces sociétés adoptent diverses approches pour faciliter le traitement de l'hépatite C, qui reposent notamment sur des stratégies de fixation des prix, l'octroi volontaire de licences, un renforcement des capacités et des dons de médicaments. Nous donnons un aperçu des efforts déployés par ces sociétés pour faciliter l'accès aux médicaments permettant de soigner l'hépatite C, et proposons des actions que peuvent mener ces sociétés afin de jouer un plus grand rôle dans l'enraiement de cette épidémie: (i) donner un degré de priorité élevé aux évaluations de l'accessibilité économique; (ii) développer des stratégies d'accès au début du cycle de vie du produit; et (iii) octroyer des licences aux fabricants de médicaments génériques.

## Resumen

La hepatitis C es una epidemia global. Se estima que, en todo el mundo, hay 185 millones de personas infectadas, la mayoría de las cuales viven en países de ingresos bajos y medios. Los recientes avances en el desarrollo de antivirales han producido terapias más efectivas, seguras y de mejor tolerancia que los tratamientos para la enfermedad existentes. Estas terapias presentan una oportunidad para poner freno a la epidemia, siempre y cuando sean asequibles, aumentar la producción genérica de dichos medicamentos y reforzar los programas de sensibilización y detección. Las empresas farmacéuticas juegan un papel central. Se examinaron los productos comercializados, tuberías y estrategias de acceso a los medicamentos de veinte de las mayores empresas farmacéuticas del mundo. Seis de estas empresas están desarrollando medicamentos para tratar la hepatitis C: AbbVie, Bristol-Myers Squibb, Gilead, Johnson & Johnson, Merck & Co. y Roche. Estas empresas emplean una gama de enfoques para apoyar el tratamiento para la hepatitis C, incluyendo las estrategias de fijación de precios, la concesión voluntaria de licencias, la creación de capacidad y las donaciones de medicamentos. Ofrecemos una visión general del compromiso de estas empresas a la hora de ofrecer acceso a los productos para tratar la hepatitis C. Sugerimos acciones que las empresas pueden llevar a cabo para tener un papel más importante a la hora de frenar esta epidemia: (i) dar prioridad a los criterios de asequibilidad; (ii) desarrollar estrategias de acceso al principio del ciclo de vida del producto; y (iii) ofrecer licencias a los fabricantes de medicamentos genéricos.

## ملخص

إن التهاب الكبد الفيروسي "سي" وباء منتشر على مستوى العالم. وتشير تقديرات عدد المصابين بهذا المرض في أنحاء العالم إلى ما يقارب 185 مليون شخص، يقيم معظمهم بالدول منخفضة ومتوسطة الدخل. وقد أثمر التقدم الذي تم إحرازه مؤخرًا في تطوير العقاقير المضادة للفيروسات عن علاجات أكثر فاعلية وأمانًا مع قابلية أعلى لتحملها من جانب المريض مقارنةً بالعلاجات الحالية لهذا المرض. وتتيح هذه العلاجات فرصة الحد من ذلك الوباء، شريطة أن تتوفر بأسعار معقولة، وأن يزداد معدل إنتاج هذا النوع من الأدوية، بالإضافة إلى تعزيز برامج التوعية والفحص. وتلعب شركات المستحضرات الطبية دورًا محوريًا في ذلك. وقد بحثنا في المنتجات التي تم تسويقها وقنوات توفير الأدوية، واستراتيجيات تيسير الحصول عليها لدى 20 شركة من ضمن أكبر شركات المستحضرات الطبية في العالم، فوجدنا ست شركات منها تعمل حاليًا على تطوير أدوية علاج التهاب الكبد الفيروسي "سي"، وهي AbbVie، وBristol-Myers Squibb، وGilead، وJohnson & Johnson، وMerck & Co.‎‎، وRoche. وتتبنى هذه الشركات مجموعة من المناهج لدعم علاج التهاب الكبد الفيروسي "سي"، بما يشمل استراتيجيات التسعير، ومنح التراخيص الاختيارية، وبناء القدرات، والتبرع بالعقاقير. كما نُجري مراجعة لمدى مشاركة تلك الشركات في توفير سبل الحصول على المنتجات المتعلقة بالتهاب الكبد الفيروسي "سي". وفي هذا الصدد، فإننا نقترح بعض الإجراءات التي يمكن أن تتخذها الشركات كي تلعب دورًا أكثر أهمية في الحد من هذا الوباء: (1) إعطاء الأولوية للتقييمات المتعلقة بمعقولية الأسعار؛ و(2) وضع استراتيجيات لتيسير الحصول على الأدوية في مرحلة مبكرة من دورة حياة المنتج؛ و(3) منح التراخيص للجهات المصنِّعة لهذه الأنواع من الأدوية.

## 摘要

丙型肝炎是一种全球性流行病。据估计，全世界约有 1.85 亿人受到感染，其中大多数人居住在中低收入国家。目前，抗病毒药物研制取得了新进展，推出了新疗法，该疗法比现有的疾病疗法更加有效、安全且耐受性更高。如果这些疗法价格实惠、这些药物的仿制药生产规模扩大并且公众意识和筛查项目增强，此类疗法就为抑制该流行病提供了一种可能。

制药公司在其中发挥着核心作用。

我们调查了世界上最大的 20 家制药公司销售的产品、渠道和药物可及性策略。其中六家公司正在研制丙型肝炎药物：艾伯维 (AbbVie)、百时美施贵宝 (Bristol-Myers Squibb)、吉利德 (Gilead)、美国强生 (Johnson & Johnson)、默克集团 (Merck & Co) 和罗氏 (Roche)。这些公司采用一系列的方法支持丙型肝炎治疗，包括定价策略、自愿授权、能力建设和药物捐赠。我们概述了这些公司在解决丙型肝炎产品可及性方面的承诺。我们为这些公司推荐了以下可行措施，它们可采取这些措施在抑制该流行病中发挥更大的作用：一、将负担能力评估放在首位；二、在产品生命周期中及早制定药物可及性策略；三、向仿制药制造商提供授权。

## Резюме

Гепатит С — заболевание уровня мировой эпидемии. По оценкам специалистов, в мире гепатитом C заражены 185 миллионов человек. Большей частью они проживают в странах с низким и средним уровнем дохода. Недавний прогресс в деле разработки антивирусных препаратов позволил создать новые методы лечения, которые эффективнее, безопаснее и переносятся лучше, чем традиционные способы лечения данного заболевания. Эти новые методы дают возможность ограничить распространение эпидемии, однако они должны быть доступны, а масштаб производства дженериков должен быть расширен. Кроме того, нам требуется укрепить программы по скрининговому обследованию и осведомленности о болезни. Центральная роль в этом процессе отводится фармацевтическим компаниям. Нами было проведено изучение имеющихся на рынке препаратов, ассортимента продукции и стратегий по обеспечению доступности лекарственных средств, выпускаемых 20 крупнейшими фармацевтическими компаниями мира. Шесть из них разрабатывают лекарства от гепатита С: AbbVie, Bristol-Myers Squibb, Gilead, Johnson & Johnson, Merck & Co. и Roche. Эти компании используют разнообразные подходы в вопросах терапии гепатита С, включая ценовые стратегии, добровольное лицензирование, наращивание мощностей и предоставление лекарств на благотворительной основе. Мы предоставляем обзор того, каким образом эти компании вовлечены в решение вопроса о доступности препаратов, предназначенных для лечения гепатита С. Мы предлагаем компаниям, которые могут играть более значимую роль в ограничении распространения этой эпидемии, принять следующие меры: (i) поставить на первое место оценку доступности препаратов, (ii) разработать стратегии по обеспечению доступности препарата на ранних этапах жизненного цикла товара и (iii) предоставить лицензии производителям дженериков.

## Introduction

Hepatitis C is an infectious disease caused by the hepatitis C virus (HCV). There is a global epidemic of hepatitis C, with approximately 185 million people estimated to be infected in 2005^1^ and 350 000–500 000 deaths estimated annually.^2^ Over 80% of those affected by the disease live in low- and middle-income countries, especially in central, north and west Africa.^3^^,^^4^ Several middle-income countries such as Egypt, Nigeria and Pakistan have a high burden of hepatitis C.

HCV is most often spread when infected blood enters the body. High-risk populations include intravenous drug-users and recipients of blood transfusions in poorly controlled environments;^2^ but the virus is also found in the general population. There are six genotypes of HCV, with distributions varying by region. It is possible for a person to be infected with multiple genotypes; 55–85% of people will develop chronic infection and about one third of these, if not treated, will eventually develop liver cirrhosis or hepatocellular carcinoma.^2^ Approximately 80% of newly-infected people are asymptomatic, which makes it difficult to diagnose and treat those who go on to develop chronic infection.^2^

Newly-available drugs have revolutionized treatment.^5^ Previous treatments were poorly tolerated and had limited success. In contrast, the new treatments are easier to administer with shorter course durations and higher cure rates.^5^

There are significant similarities between the hepatitis C epidemic and the human immunodeficiency virus (HIV) crisis of the late 1990s. Both involve global spread of underdiagnosed disease that can carry significant stigma and cause life-long illness and death. In both cases, there are new, effective products that can curb the global epidemic, held back by issues of affordability. The HIV crisis in South Africa resulted in a clash between civil society, government and the pharmaceutical industry,^6^ which led to the development of new ways of working, most notably with generic medicine manufacturers via licensing arrangements, creating a blueprint for access to medicine strategies for years to come.

However, there are also critical differences between the two epidemics that influence how pharmaceutical companies design access strategies. With HIV, the greatest disease burden is situated in lower-income countries and concentrated in sub-Saharan Africa.^7^ These countries were not viewed, in the earlier days of the epidemic, as representing market potential. In contrast, HCV is prevalent in some middle-income countries.^4^ Middle-income countries, though home to most of the world’s poor,^8^ have growing middle classes,^9^ representing economic opportunities for pharmaceutical companies. As a result, companies have an incentive to maintain exclusivity and charge higher prices in these markets. This may discourage donor, insurer and government investment in expensive new treatments,^10^ potentially slowing the adoption of new medicines.

Here we identify marketed products, product pipelines and access to medicine strategies of companies that make HCV medicines. We describe companies’ plans and activities to support greater access to HCV treatment and set out the criticisms, limitations and opportunities of these approaches. Finally, we present recommendations for companies to consider when developing access to medicine strategies for HCV-infected people.

## Hepatitis C medicines

We used data from the *Access to Medicine Index 2014*^11^ and other publicly-available sources such as company, patients’ organization and nongovernmental organization websites. The access to medicine index is created by the Access to Medicine Foundation, an independent initiative funded by the Bill & Melinda Gates Foundation, the Dutch Ministry of Foreign Affairs, the United Kingdom Department for International Development, and the Dutch National Postcode Lottery. The Access to Medicine Foundation engages directly with 20 of the world’s largest pharmaceutical companies, requesting data biennially on selected activities via a detailed online questionnaire.^12^ The data are used to rank the companies in a biennial index. This index uses a set of 95 indicators to assess companies’ comparative performance in facilitating access to medicines in poor populations. A limitation of the data is that they are largely self-reported by companies. However, the data are reviewed by an external research partner (for the 2014 index, this partner was Sustainalytics, Amsterdam, Netherlands) and the foundation’s research team, clarified with companies and verified in some areas with external data sources.

### Current treatment

The World Health Organization (WHO) released new treatment guidelines for hepatitis C infection in April 2014.^1^ Given that most patients will not realize they are infected, screening is recommended for high-risk groups. In countries with high prevalence and low infection control, screening is recommended for the whole population, if resources allow this.^1^ However, diagnostic capacity is limited in many low- and middle-income countries.^10^

The size of the population requiring treatment for hepatitis C is difficult to gauge. As noted, not all of the 185 million people estimated to be infected will progress to chronic infection, and there are no conclusive predictors of disease progression.^13^ There is currently no vaccine against HCV.^1^ In higher-income countries where treatment is available, all persons diagnosed with chronic HCV infection are typically considered suitable for treatment. In countries where treatment availability is constrained, treatment is prioritized for patients in more advanced stages of the disease. Different treatment regimens are advised depending upon HCV genotype.^1^

Older HCV treatments comprised combination antiviral therapy with pegylated interferon (weekly injections) and ribavirin (tablet, capsules, or oral solution). Pegylated interferon, which remains on patent in most countries, was added to WHO’s essential medicines list in 2013. Ribavirin is off-patent and generic versions exist. Two companies (Merck & Co, Kenilworth, United States of America (USA) and Roche, Basel, Switzerland) included in the access to medicines index manufacture interferon; one also manufactures ribavirin (Roche). This treatment regime is not widely available, can be poorly tolerated and has undesirable side-effects. In Egypt, a 48 week course of peginterferon/ribavirin costs 2000 United States dollars (US$).^1^ Only 30–50% of people are cured, partly because many patients don’t finish treatment.^1^^,^^2^

### New treatment options

New antiviral drugs for HCV infection, known as oral directly-acting antiviral agent therapies, are now available on the market. They are more effective, safer and better-tolerated than existing therapies: 90% of people are cured.^14^ The therapies are orally administered and have shorter treatment courses (12–24 weeks depending on regimen and genotype),^1^ which decreases monitoring requirements.^5^ Five currently-marketed therapies were added to WHO’s essential medicines list in 2015 (Table 1).^15^

**Table 1:** Currently marketed hepatitis C medicines from pharmaceutical companies, 2015

Company	Brand name	INN	Class	Market approval		EML
				FDA	EMA	
AbbVie	Viekira Pak®	ombitasvir/paritaprevir/ritonavir + dasabuvir	Direct acting antiviral (combination)	2014	–	No
AbbVie	Viekirax®	ombitasvir/paritaprevir/ritonavir	Direct acting antiviral	–	2015	Yes
AbbVie	Exviera®	dasabuvir	Direct acting antiviral	–	2015	Yes
Bristol-Myers Squibb	Daklinza®	daclatasvir	Direct acting antiviral	–	2014	Yes
Gilead	Sovaldi®	sofosbuvir	Direct acting antiviral	2013	2014	Yes
Gilead	Harvoni®	sofosbuvir/ledipasvir	Direct acting antiviral (combination)	2014	2014	Yes
Johnson & Johnson	Olysio®	simeprevir	Direct acting antiviral	2013	2014	Yes
Johnson & Johnson	Incivo®	telaprevir	Direct acting antiviral	2011^a^	2011	No
Merck & Co.	Victrelis®	boceprevir	Direct acting antiviral	2011^a^	2011	No
Merck & Co.	PegIntron®	peginterferon alfa-2b	Interferon	2001	2000	Yes
Roche	Pegasys®	peginterferon alfa-2a	Interferon	2002	2002	Yes
Roche	Copegus®	ribavirin	Nucleoside analogue	2002	2002	Yes

EMA: European Medicines Agency; EML: World Health Organization Essential Medicines List; FDA: US Food and Drug Administration; INN: international nonproprietary name.

^a ^Now discontinued in the US market.

Note: Only FDA and EMA drug registrations are tracked.

Of the 20 companies evaluated in the access to medicines index, six are active in HCV medicine development (AbbVie, Chicago, USA; Bristol-Myers Squibb, New York City, USA; Gilead, Foster City, USA; Johnson & Johnson, New Brunswick, USA, Merck & Co., Roche). All have products on the market and in development. In the US market Merck & Co. and Vertex recently discontinued boceprevir and telaprevir respectively, with Merck & Co. citing “advancement in treatment practices”.^16^ Boehringer Ingelheim (Ingelheim am Rhein, Germany) ceased engagement in research and development for HCV in June 2014, in view of “multiple drug approvals expected from alternative manufacturers”.^17^

One company that is not included in the access to medicines index (Achillion, New Haven, USA) was identified as active in HCV drug development; Genentech (San Francisco, USA), Kadmon and Medivir AB (Stockholm, Sweden) are also involved in marketing HCV products.^18^

**Fig. 1:**
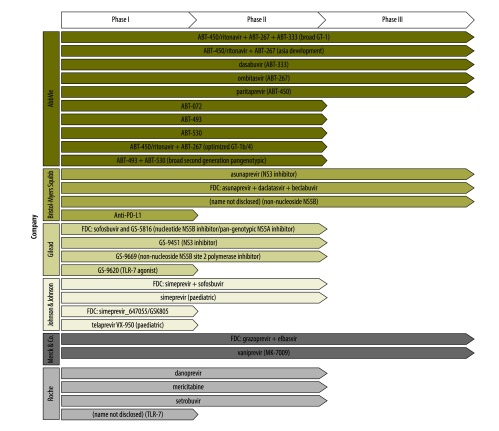
Clinical trial stage of 32 hepatitis C medicines, 2015

FDC: fixed-dose combination; GT: genotype; NS3: nonstructural protein 3; NS5A: nonstructural protein 5A; NS5B: nonstructural protein 5B; PD-L1: programmed death-ligand 1; TLR: toll-like receptor.

Note: Codes appearing within this table (e.g. ABT-450) are company codes, used to identify the drug while it is in development.

Table 1 and Fig. 1 show, respectively, currently marketed HCV products (FDA and EMA registrations only) and an overview of products in research and development, up to date at the time of this paper’s submission. Table 1 indicates the presence of several new products on the market. Fig. 1 summarizes the progress in development of HCV medicines, with many products in phase III of clinical trials. Future competition may play an important role in enhancing affordability, contingent on the relative efficacy of competitors and how broadly companies choose to register them.

### Access to medicine strategies

For the 2014 access to medicine index, companies were asked to disclose plans for making HCV products available in low- and middle-income countries. We refer to these as company access strategies. Companies shared information about equitable pricing strategies (which take affordability into account for poor populations), voluntary licensing agreements, donations of products and capacity-building initiatives. Johnson & Johnson and AbbVie disclosed no access to medicine strategies. Several new HCV products and access strategies were launched after the period of analysis ended. A summary of the access strategies identified is provided in Table 2.

**Table 2:** Access strategies for hepatitis C medicines, 2015

Company^a^	Brand name	Pricing	Financing	Licensing	Capacity building^b^	Donations
AbbVie	Viekira Pak®	No	No	No	No	No
Bristol-Myers Squibb	Daklinza®	C	No	C	Yes	No
Gilead	Sovaldi®	Yes	No	Yes	No	No
Johnson & Johnson	Olysio®	No	No	No	No	No
Merck & Co.	Pegintron®	Yes	Yes	No	Yes	No
Roche	Pegasys® and Copegus®	Yes	No	No	Yes	Yes

^a^ With marketed product or products.

^b^ Including philanthropic activity.

Note: Yes: has a strategy; No: does not have a strategy; C: commitment only

Disclosure of access to medicine strategies for products that were still under development was limited across in-house and collaborative projects. This may reflect intense competition in HCV medicines. Only Gilead disclosed access strategies for products under development, having agreed licensing conditions for two products before registration.

Access strategies are in place for two newly marketed products: sofosbuvir and ledipasvir (Gilead) and were also in place for boceprevir (Merck & Co, now discontinued). Three companies are active in pricing strategies (Gilead, Merck & Co. and Roche). Only Gilead is currently actively licensing HCV products. In late 2014, Bristol-Myers Squibb announced an intention to engage in both licensing for HCV products and tiered pricing (in which different price points are set depending on the market in which the product is sold).^19^

## Discussion

Ensuring access to medicines is a joint responsibility of governments, companies, multilateral agencies and nongovernmental organizations (NGOs). Pharmaceutical companies, being private entities, must also be able to justify their access to medicine activities to shareholders.

We have highlighted two key points of entry for pharmaceutical companies to help to address access to HCV medicines. First, affordability: the high prices attached to new medicines may be deterring donors (international or bilateral), health insurers and governments from committing sufficient funds to curbing the epidemic.^10^ Where drugs are paid for out-of-pocket, ensuring affordability is also critical. Second, generic manufacturers can scale up and distribute new medicines. Generating sufficient competition among generic manufacturers will place downward pressure on prices.

### Affordability

Manufacturers of new HCV medicines will benefit from market exclusivity until around 2025.^10^ For Gilead, this monopoly has been magnified by the dominance of sofosbuvir. However, this lead will be increasingly challenged as other new entrants compete for market share. This effect is already being seen as prices for sofosbuvir in the United States are being increasingly discounted in the face of competition.^20^

Companies should develop, with governments, mechanisms for significant price discounting based on rigorous, well researched, transparent assessments of affordability in low- and middle-income countries, clearly taking account of the needs and abilities of payers and the presence or absence of subsidies.

Separate pricing policies within countries can make sound business sense and improve access.^21^ In middle-income country markets with greater economic value to companies, private and public systems can be offered different brands and prices. This is known as market segmentation. Proposed strategies from Gilead for enforcing market segmentation of sofosbuvir requiring patient identification and limited dispensing were criticized for risking confidentiality and adherence.^22^ For poorer populations, high-volume, low-cost approaches may prove more profitable: in the United States, Gilead appears to be shifting to a higher-volume/lower-price model as competition from AbbVie intensifies.^23^

Gilead initially charged US$ 84 000 for a course of sofosbuvir in the United States. The lowest identified price for that treatment in a developing country was US$ 900 in Egypt.^24^ These prices are much higher than the drug’s production cost, which is US$ 68 to US$ 136 for a 12-week course.^25^ We were not able to estimate the research and development costs for this drug, and Gilead states publicly that they do not track this expenditure per product.^26^ High prices may be deterring bilateral, international and national funders from allocating funding at the scale required for the widespread rollout of new HCV treatments.

### Generic partners

The lessons from scaling up HIV/AIDS medicines have shown that the capacity of generic medicine manufacturers to produce high-quality medicines and their knowledge of local regulatory requirements are important for manufacture, distribution and treatment. Equally important is the impact of sufficient competition from generic medicine manufacturers on prices: the average price of a first-line adult antiretroviral regimen dropped from US$ 414 per person per year in 2003 to US$ 74 in 2008.^27^

Licensing can also make business sense. For example, efficiencies may exist in engagement with generic manufacturers who understand local regulatory requirements well, have an existing network of contacts with developing country governments and regulators and can rapidly scale up production. Manufacturers may derive income from the application of royalties to licence agreements, for example 7% royalties were attached to the licences agreed by Gilead for sofusbuvir.^28^

To date, Gilead is the only manufacturer of new HCV medicines to have completed agreements with generic medicine manufacturers. The number of manufacturers^12^ is, arguably sufficient to engender competition and the full agreements have been publicly disclosed.^29^ Although the scope of the licences appears broad, they do not include key middle-income country markets with high HCV burdens, such as Brazil, China, Georgia, Mexico, Thailand and Ukraine.^30^ Further, it is not yet clear on what scale generic manufacturers will enter the market, nor what discounts will be realized, though NATCO, Hyderabad, India, has disclosed a price of 19 900 Indian rupees for 28 400 mg tablets in Nepal.^31^ On a 12-week course of treatment, this equates to approximately US$ 900. So, although Gilead’s licensing activities represent leading practice among the companies included in the access to medicine index, this approach has limitations in geographic scope and improvements in affordability are not yet clear. Bristol-Myers Squibb has also announced its intention to engage with generic medicine manufacturers, detailing the geographic scope of future licences.

Regarding licensing, it is important that pharmaceutical companies agree to the most flexible terms possible, for example, minimizing royalties, not placing restrictions on supply of active pharmaceutical ingredients and allowing supply to as broad a range of countries as possible. This provides generic medicine manufacturers with the greatest potential to compete and keep prices low.

Market segmentation can broaden the geographic scope of licences. Where patent-holders may wish to retain monopoly over higher income segments, licences can limit markets generic manufacturers can sell to. GlaxoSmithKline and Pfizer, via their joint-venture ViiV Healthcare, have tested this approach in the context of HIV medicines with the Medicines Patent Pool, segmenting public and private markets for dolutegravir and introducing a tiered royalty structure that enabled the inclusion of more middle-income countries.^32^

### Plan access strategies early

Research-based companies should consider developing access strategies such as equitable pricing or voluntary licensing earlier in a product’s lifecycle (for example, in the later stages of research and development). This could reduce the time taken for products to reach those in need. In the case of voluntary licensing, agreeing terms with generic manufacturers early extends the time available for technology transfer, thereby enabling licencees to start production as soon after product registration as possible. Gilead has licensed medicines before product registration.^33^ To our knowledge, no other pharmaceutical company has engaged in pre-registration licensing for HCV products.

### Supporting awareness and diagnosis

Since most HCV infections are initially asymptomatic, it is also important to raise sufficient awareness, reduce stigma, and build screening and diagnostic capacity to curb the epidemic. In addition to pricing and licensing, some companies disclosed details of programmes for supporting local screening and diagnostic capacity. It should be noted that such activities bring with them a significant risk of conflict of interest associated with direct contact with patient organizations or health-care professionals. Companies may seek to manage the risk of conflict through the involvement of established NGOs and/or WHO, aligning with the needs of ministries of health and integrating with existing programmes.

Merck & Co. is building screening capacity in Latin America, aimed at reducing the time taken for results to be received. Likewise, Roche, with strength in diagnostics, is engaged in building screening capacity in central Europe, the Eastern Mediterranean region and India, including awareness-raising activities. Bristol-Myers Squibb is engaged in a project aimed at raising awareness in China, India and Japan.

## Conclusion

It is clear that treatment of hepatitis C is undergoing a revolution. It is not enough however, to develop effective treatment. All actors in the global health community need to ensure that these new products are available, accessible and affordable for all in need. This goal ultimately requires a multi-actor, multi-pronged approach.

The lack of access strategies disclosed for products under development is concerning, especially those in phase III clinical trials, which bear the greatest chance of market entry. It is also concerning that some companies disclosed no access strategies for either currently marketed products or products in the pipeline.

As described, pharmaceutical companies have central roles to play, particularly with regard to ensuring affordability and voluntary licensing. The available evidence provided by companies so far suggests a need for more concerted, broader engagement in access strategies.
